# Prothonotary warbler nestling growth and condition in response to variation in aquatic and terrestrial prey availability

**DOI:** 10.1002/ece3.2400

**Published:** 2016-09-28

**Authors:** Jenna C. Dodson, Nicholas J. Moy, Lesley P. Bulluck

**Affiliations:** ^1^ Center for Environmental Studies Virginia Commonwealth University Richmond Virginia; ^2^ Department of Biology Virginia Commonwealth University Richmond Virginia

**Keywords:** Aquatic prey, food availability, migratory bird, phenology, prey subsidies

## Abstract

Aquatic prey subsidies entering terrestrial habitats are well documented, but little is known about the degree to which these resources provide fitness benefits to riparian consumers. Riparian species take advantage of seasonal pulses of both terrestrial and aquatic prey, although aquatic resources are often overlooked in studies of how diet influences the reproductive ecology of these organisms. Ideally, the timing of resource pulses should occur at the time of highest reproductive demand. This study investigates the availability of aquatic (mayfly) and terrestrial (caterpillar) prey resources as well as the nestling diet of the prothonotary warbler (*Protonotaria citrea*) at two sites along the lower James River in Virginia during the 2014 breeding season. We found large differences in availability of prey items between the two sites, with one having significantly higher mayfly availability. Nestling diet was generally reflective of prey availability, and nestlings had faster mean growth rates at the site with higher aquatic prey availability. Terrestrial prey were fed more readily at the site with lower aquatic prey availability, and at this site, nestlings fed mayflies had higher mean growth rates than nestlings fed only terrestrial prey. Our results suggest that aquatic subsidies are an important resource for nestling birds and are crucial to understanding the breeding ecology of riparian species.

## Introduction

Food availability is a main determinant of reproductive success in animals (Daan et al. 1989; Tremblay et al. 2003), and this is especially true in altricial species that require a high degree of parental care (Brinkhof and Cave 1997). Resource availability may influence reproductive success via multiple mechanisms. For example, studies in insectivorous songbirds have shown that food availability is correlated with egg size (Ardia 2006) and the number of young produced (Nagy and Holmes 2005b). Faster nestling growth rates have also been found in habitats with greater invertebrate biomass (Duguay et al. 2000; Naef‐Daenzer et al. 2000) or in artificially food‐supplemented nests (Simons and Martin 1990; Brinkhof and Cave 1997). Studies of food availability in songbirds have focused primarily on species with a relatively simple terrestrial diet (e.g., caterpillar specialists), likely due to the logistical challenges of simultaneously sampling multiple prey types. However, the flux of adult aquatic insects into riparian habitats can provide a considerable dietary subsidy for terrestrial predators as varied as spiders (Burdon and Harding 2008), birds (Nakano and Murakami 2001), bats (Sullivan et al. 2014), and lizards (Sabo and Power 2002). Despite the fact that aquatic prey can account for a significant proportion (up to 90%) of the energy budget for some bird and spider species (Nakano and Murakami 2001; Iwata et al. 2003), studies of how these fluxes of aquatic prey affect fitness measures (i.e., growth and survival) of terrestrial consumers are rare (but see Sabo and Power 2002 and Strasevicius et al. 2013).

Reproductive success can be affected by the timing of breeding with regard to seasonal resource availability (Dias and Blondel 1996; Seki and Takano 1998; Naef‐Daenzer et al. 2000; García‐Navas and Sanz 2011). Ideally, the energy‐demanding nestling stage should coincide with seasonal resource peaks (Rossmanith et al. 2007). This idea has been extensively studied in two nonmigratory caterpillar specialists, Great and Blue Tits (*Parus major, Parus caeruleus)* in Europe where the timing of breeding was found to be synchronized with the spring peak in caterpillar abundance (Van Noordwijk et al. 1995; Dias and Blondel 1996; Seki and Takano 1998; Naef‐Daenzer et al. 2000; Tremblay et al. 2003). The degree of synchronization between laying date and caterpillar peak positively affects fledgling size and number (Van Noordwijk et al. 1995; Dias and Blondel 1996; Tremblay et al. 2003). However, many migratory species arrive during the peak in caterpillar resources and therefore do not show a strong link between the timing of nestling provisioning and seasonal resource peaks (Marshall and Cooper 2004; Maziarz and Wesolowski 2010). One hypothesis is that these species may rely on early peaks in caterpillar resources for egg production and other prey resources to rear their young (Daan et al. 1989). In riparian breeding species, these other prey resources are likely emerging aquatic insects, although few studies have quantified the timing of avian reproduction relative to the seasonal pulse(s) in aquatic prey. Riparian species that optimize the timing of breeding to coincide with such pulses may increase their reproductive success.

In this study, we examine variation in aquatic and terrestrial food resources of the prothonotary warbler (*Protonotaria citrea*), a riparian migratory songbird, and how variation in these resources affects nestling diet, growth, and condition. Specifically, we quantified the temporal variation in caterpillar (Lepidoptera) and mayfly (Ephemeroptera) biomass over one breeding season at two study sites in eastern Virginia. These two prey items were observed in previous breeding seasons to be the primary prey items brought to nestlings at one of our study sites (L. Bulluck, pers. obs.). Our second objective was to determine whether variation in terrestrial and aquatic prey availability influences prothonotary warbler nestling provisioning. Lastly, we assessed whether diet was correlated with nestling growth rate and body condition.

## Materials and Methods

### Site and study species

Prothonotary warblers are Neotropical migratory birds that nest in bottomland hardwood forests throughout the southeastern United States. Their nests are usually in cavities near or over water, and as such, they use aquatic and terrestrial prey resources. The most common prey items include caterpillars and spiders (terrestrial), and mayflies and midges (aquatic) (Petit et al. 1990a). Prothonotary warblers are cavity nesters and will readily nest in artificial nest boxes, making them an ideal species to study their reproductive ecology and diet.

A long‐term study of prothonotary warblers breeding in nest boxes along the lower James River and its tributaries began in 1987 (Blem et al. 1999). This study focused on two sites: Presquile National Wildlife Refuge (Presquile NWR) and Deep Bottom Park in Henrico County, Virginia. Deep Bottom Park is approximately 8 km upstream from Presquile NWR. The nest boxes are situated near the shoreline along the main stem of the river and in a smaller tributary (mean width = 20 m). River width from the mouth of the tributary at Deep Bottom Park is 185, and 325 m at Presquile NWR. Both sites are a combination of tidal freshwater swamp and bottomland hardwood forest, with green ash (*Fraxinus pennsylvanica*), red maple (*Acer rubrum*), sycamore (*Platanus occidentalis*), oak (*Quercus* spp.), black gum (*Nyssa sylvatica*), and hornbeam (*Carpinus caroliniana*) as the dominant tree species. Both sites are situated on river meanders; however, the site at Presquile has notably more sedimentation around the oxbow and deposition throughout the creek compared with Deep Bottom. This is likely the result of a channel cut made in 1934 at the base of the oxbow that turned Presquile NWR into an island.

### Nestling field surveys

A total of 110 nest boxes were monitored in 2014: 63 boxes at Deep Bottom Park and 47 boxes at Presquile NWR. Mean yearly temperature and total precipitation were consistent with climate data for this area (mean 15°C and 90.7 cm total rainfall). All boxes were positioned on metal conduit poles approximately 30 m apart over water at high tide and were checked 2–3 times each week during the breeding season (late April–mid‐July). Nest contents were recorded to determine dates for nest initiation (first egg), hatching, and completion for each clutch. As a species and population that often double broods (Bulluck et al. 2013), there is a natural lull in hatching dates during the season between the early and later clutches. This lull occurred on May 28 such that nests with eggs that hatched prior to this date were classified as early clutches, and those nests whose eggs hatched after 28 May were classified as late clutches.

**Figure 1:**
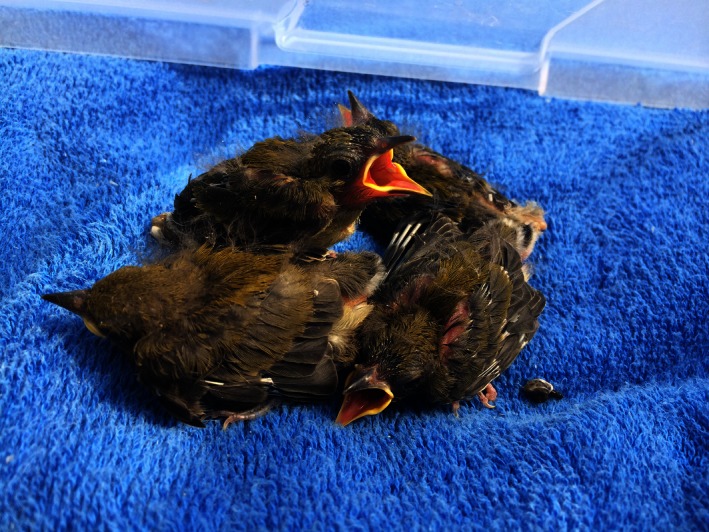
Nine‐day‐old prothonotary warbler nestlings removed from their nest box for measuring (photograph taken by L. Bulluck).

Nestlings were carefully removed from the nest box (Fig. 1) and fitted with a unique numbered aluminum band (USGS). We measured the mass and tarsus length of each nestling on two occasions between hatching and fledging (mean age of first weighing was 3–4 days, range 2–8 days, mean age of second weighing was 7–8 days, range 5–10 days, with hatch day = 0). We were not able to standardize the ages for these measurements due to the logistics of accessing the sites and boxes. Tarsus length was measured to 0.1 mm using dial calipers, and mass was measured to 0.1 g using a digital scale. Mean growth rates per brood were calculated as the change in mean mass between the first and second weighing dates, standardized by the number of days between measurements. Nestling body condition was calculated as the residuals from a regression of tarsus and mass at the second measurement, and was calculated for each age class separately (Schulte‐Hostedde et al. 2005). All applicable institutional and/or national guidelines for the care and use of animals were followed. All research was approved by the Virginia Commonwealth University's Institutional Animal Care and Use Committee (IACUC, protocol #AM10230) as well as the VA Department of Game and Inland Fisheries (permit #053965) and the US Department of Interior (USGS Bird Banding Laboratory permit #23486).

### Sampling of caterpillar availability

To estimate caterpillar abundance, we used a branch‐clipping apparatus and methods described by Johnson (2000). Branch clipping was conducted weekly in 15‐m‐radius plots along near‐shore transects. Each week we collected three branch clippings, standardized by leaf mass, from four randomly selected plots (12 per site) for a total of 24 branch clippings per week between the two sites. Prothonotary warblers are generalist foliage‐gleaning insectivores (Petit et al. 1990b), and during the breeding season, both males and females forage most often below 6 m, concentrating their foraging maneuvers on the middle and outer parts of trees and shrubs (Petit et al. 1990b). We sampled the outer branches of the most common tree species in each plot at heights ranging from 0.5 to 6 m. Within a plot, three branch clippings were taken from three different trees.

After a branch was collected, it was carefully inspected for invertebrates and all leaves were removed at the base of the petiole and collected. All invertebrates were identified to order. The primary species of caterpillars collected were Geometridae. Both insects and leaves were dried at 80°C for at least 24 h and weighed (dry mass ± 0.0001 mg). Caterpillar biomass was calculated for each branch sample as mg caterpillar dry mass·mg^−1^ leaf dry mass, and weekly averages were calculated for each site.

### Sampling of mayfly availability

We used a combination of Pennsylvania style light traps (Frost 1957) and emergence traps (Davies 1984) to sample mayfly abundance. At each site, eight emergence traps (0.86 m × 0.86 m) were placed approximately 10 m from the shore, four along the main stem of the river and four along the tributary, following the layout of the nest boxes. Emergence traps were deployed on 28 April and were checked weekly until soon after the first emergence (15 May), after which light traps were used as our primary weekly sampling method. Adult mayflies were collected from the emergence trap using a Heavy Duty Hand Held Aspirator from BioQuip (item number 2820GA). Light traps were set up 30 min prior to sunset and samples collected 2 h after sunset on evenings when there was no rain or strong wind. All collected insects were frozen until processing. All mayflies (Ephemeroptera, *Hexagenia* spp) were picked from each sample, dried in the oven at 80°C for at least 24 h, and weighed (dry mass ± 0.0001 mg). Mayfly emergence from emergence traps was quantified as total g dry mass·m^−2^ of trap area·week^−1^ to determine weekly rate of emergence. Weekly light trap samples were quantified as total mg of dry mass per hour after sunset.

### Quantifying nestling diet

Nestling diet was quantified using video observations. A Canon FS400 handheld standard‐definition camera (min/max focal length = 2.6/96.2 mm) was placed 2–4 m from the nest box with a clear view of the nest box opening for at least 1.09 h (mean video length was 2.55 h, range 1.09–3.75 h). All video observations were conducted in the mornings (6:00–12:30) when the nestlings were 6–9 days old. We observed no difference in feeding behavior in the presence of the video cameras (e.g., prolonged nest vigilance or excessive chipping around nest or camera), and cameras have been successfully used to quantify nestling provisioning in other warbler species (Stodola et al. 2010). Videos were reviewed by four observers for the identification of food items brought to the nest. All observers were initially trained by watching and scoring the same video and then discussing any interobserver differences, and regular meetings were held to discuss identification of any questionable prey items. For each visit, we recorded the number and size of food item(s) as well as parent sex using plumage characteristics and color band combinations. When possible, food items were identified to one of the following orders: Lepidoptera (caterpillar – terrestrial), Ephemeroptera (mayfly – aquatic), Araneae (spider – terrestrial), Orthoptera (grasshopper – terrestrial), Odonata (dragonfly – aquatic), other terrestrial (i.e., Coleoptera larvae or pupae), or unknown.

The length (size) of food was estimated relative to the parents' bill (1 = smaller than bill, 2 = same size as bill, 3 = larger than bill) following Beck (2010) and Stodola et al. (2010). These size estimations were used to calculate a food score. A food score was calculated for each visit as the food size multiplied by the number of items. These food scores were totaled for each prey type and for each nest. If the item size could not be estimated because the item was too small (e.g., inside the beak), it received a size of one. If an item could not be identified due to visual obstruction, it was classified as a nonvisualized unknown prey item and did not receive a size score. In these cases, we assumed that nonvisualized prey items were consistent with the sizes of identified items, and calculated an adjusted food score estimate. To calculate the adjusted food score estimate, the number of visits with nonvisualized prey items was multiplied by the average food score per visualized item for that parent. These values provided an estimated food score of the nonvisualized unknown item(s). This was performed for males and females separately and added to the total food score, creating a total adjusted food score for that nest. When an item was not visualized but a parent displayed normal feeding behaviors such as perching on the box entrance and lowering its head and neck into the box, it was assumed one item was being fed. All diet variables were standardized by number of nestlings and video length (chick^−1^·h^−1^).

### Statistical analyses

We compared weekly caterpillar biomass between sites using a Wilcoxon rank‐sum test because the assumption of normality was not met (zero‐inflated distribution). The amount of food brought to nestlings (food score chick^−1^·h^−1^) was compared among nestling age classes using a one‐way analysis of variance (ANOVA), and we assessed whether video start time influenced the amount of food brought to nestlings or the number of parental visits (chick^−1^·h^−1^) using simple linear regression. Assumptions of normality were met for these analyses based on Shapiro–Wilk tests (total food score *w* = 0.981, *P* = 0.166, total visits *w* = 0.992, *P* = 0.814).

In order to assess the influence of site, date, and weekly availability on the prey types brought to nestlings, we conducted a multivariate analysis of variance (MANOVA) that accounted for the interdependence of different prey types being fed (García‐Navas and Sanz 2011). Specifically, the caterpillar and mayfly food scores at a nest are not independent, and the MANOVA tested for changes in prey type simultaneously as well as for univariate effects on prey types individually. The dependent variables in the MANOVA were mayfly food score chick^−1^·h^−1^ and caterpillar food score chick^−1^·h^−1^. The independent variables were site, date, and the relative site availability of these prey items the week that the provisioning video was taken; caterpillar and mayfly availability were calculated from branch clippings and light trap samples, respectively. No mayflies were captured in the vicinity of nest boxes at Presquile despite weekly sampling efforts, yet some nestlings were fed mayflies at this site (see Results). We therefore assume that mayflies also peaked in the same week near Presquile such that nests were assigned the weekly mayfly availability values from Deep Bottom in the MANOVA. We also tested for an interaction between site and date on the type of prey delivered to nests.

Prior to analyses, weekly mayfly biomass, weekly caterpillar biomass, and nest‐level provisioning food scores were log_10_ (*x* + 1) transformed to improve normality. The transformation led to normal distributions for Deep Bottom food score values (Shapiro–Wilk test *P* > 0.05) but not nest‐level mayfly food scores at Presquile (see Results) nor caterpillar and mayfly biomass collected each week to assess prey availability. However, because MANOVA is robust to violations of the normality assumptions when the sample sizes are large (nest‐level mayfly food scores *n *=* *40), we present the results but do not plot the regression line for the non‐normal data (Presquile mayfly food scores, see Results) in the figure showing these results. Further, because prey availability and use between sites were so different (see Results), we conducted subsequent analyses assessing growth rate and condition separately for each site.

To determine how diet influences mean nestling growth rate and body condition, we developed linear regression models for each site and used Akaike's information criterion (AIC_c_) adjusted for small sample size to compare the models (Burnham and Anderson 2002). All provisioning data were collected at the brood level (not for each individual nestling), and we used brood mean growth rate and body condition as the response variables in these models. Before specifically testing the effects of mayfly and aquatic (mayfly plus other emerging aquatic insect) prey, we first assessed the following factors shown in previous studies to influence nestling growth rate and/or condition: date, mean brood age, brood size, male visits chick^−1^·h^−1^, and female visits chick^−1^·h^−1^ (Neill and Holmes 1996; Podlesak and Blem 2001; Stodola et al. 2010). Brood age was excluded in the body condition model as it was calculated separately for each age. We used backward stepwise regression with growth rate or body condition as the dependent variable and AIC_c_ as the criterion for variable exclusion. The result of this stepwise procedure was then considered the base model upon which to test the hypothesis that aquatic prey influenced nestling growth rate and condition. We compared the best performing base model with models including either aquatic food score (chick^−1^·h^−1^) or mayfly food score (chick^−1^·h^−1^) using AIC_c_ and adjusted *R* ^2^ values to see whether these predictors improved model fit. Because mayfly and aquatic food score values from Presquile were zero‐inflated, we converted it to a categorical value at this site (aquatic or mayfly FS_cat_: 0 = nests fed no aquatic or mayfly prey, 1 = nests fed aquatic or mayfly prey); however, this was not necessary for mayfly and aquatic food scores at Deep Bottom (Shapiro–Wilk test *P* > 0.05). Mayfly and aquatic food score values are highly correlated with each other and are never included in models at the same time.

Lastly, we compared site‐level nestling growth rate and body condition using *t*‐tests. Due to the large differences in prey availability and use at the two sites, we also compared site‐level nestling growth rate (separating Presquile nests that were and were not fed aquatic prey) using a Kruskal–Wallis test. We compared double brooding rate using a chi‐square analysis and number of young fledged, clutch initiation date, and length of the nestling period using *t*‐tests. Eleven nests were excluded from analyses: two that were parasitized by brown‐headed cowbirds (*Molothrus ater*) at Presquile NWR, two with a brood age of ten at Presquile NWR, and seven with a brood size of one, three at Presquile NWR, and four at Deep Bottom. All analyses were carried out in JMP 11.2.0 (SAS Institute Inc. 2013). Unless otherwise stated, results are given throughout as mean ± 1 standard deviation.

## Results

### Prey availability and bird phenology

**Figure 2:**
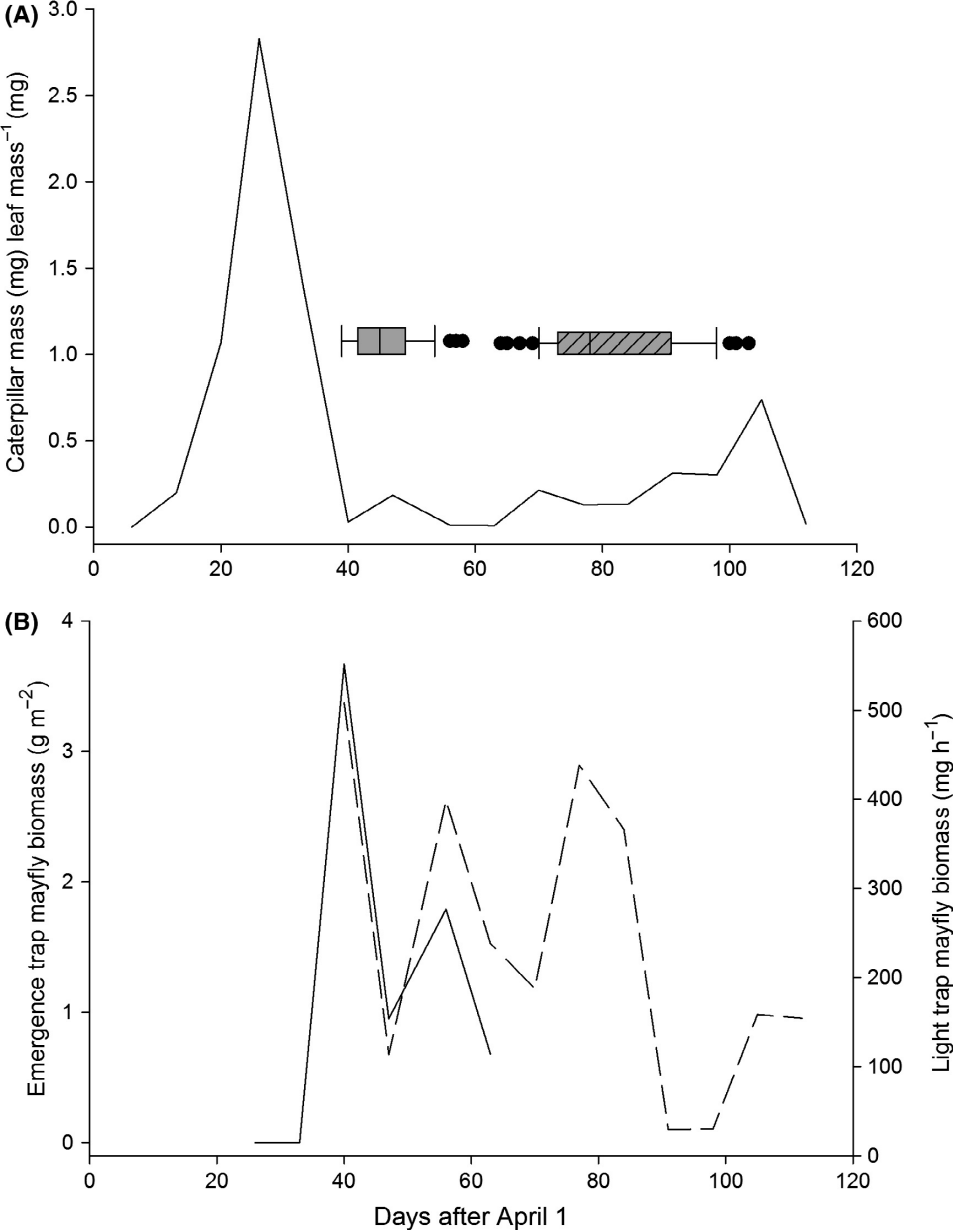
Distribution of prothonotary warbler hatching dates by clutch (box whiskers; open for early clutch, hashed for late clutch) in relation to caterpillar biomass (A) and mayfly biomass (B) from both trap types (solid line emergence traps, dashed line light traps) at Deep Bottom Park and Presquile National Wildlife Refuge (NWR) throughout the 2014 breeding season. Medians, 25th and 75th percentiles (boxes), 10th and 90th percentiles (whiskers), and outliers (dots) are shown. Caterpillar data are pooled values from both sites while mayfly data are only from Deep Bottom because no mayflies were captured at Presquile NWR.

Caterpillars were abundant at both sites, and there was no variation in weekly caterpillar biomass between sites (Deep Bottom mean = 0.72 ± 1.6 mg, Presquile = 0.22 ±0.38 mg, Wilcoxon *χ* ^2^ = 1.63, *P *=* *0.202). There were two distinct caterpillar biomass peaks, the largest peak occurred in the first week of May and the second in mid‐July, after most nestlings had fledged (Fig. 2). The day of maximum egg production at both sites (9 May, *n *=* *113 nests, 361 eggs) occurred within 1 week after the peak in caterpillar biomass. The date of maximum nestling demand (19 May, *n *=* *113 nests, 214 nestlings) occurred during a time of low caterpillar availability and caterpillar biomass remained relatively low throughout the nestling period (Fig. 2A).

Mayfly biomass differed significantly between the two sites. Despite weekly light‐trapping efforts, only one mayfly was caught in the vicinity of our nest boxes at Presquile NWR. At Deep Bottom, mayflies were abundant and captured throughout the nestling period (Fig. 2). The day of maximum egg production and hatch date of the earliest nest (9 May) occurred 3 days before mayfly emergence (12 May). The date of maximum nestling demand occurred during a time of high mayfly biomass, and mayfly biomass remained high, although variable, for the duration of the season (Fig. 2).

### Nestling diet

Video observations of nestling provisioning were recorded for 99 nests (Deep Bottom *n *=* *59, Presquile NWR *n *=* *40). Data from a total of 253 h and 2755 visits were recorded, and 73% of prey items were identified out of 3266 prey items brought to the nests. The amount of food brought to the nest did not differ among brood age classes (one‐way ANOVA, *F*_2,101_ = 2.11, *P *=* *0.127), and there was no relationship between start time of the video and the number of visits (*P *=* *0.735) or the total amount of food brought to the nest (*P* = 0.692).

**Figure 3:**
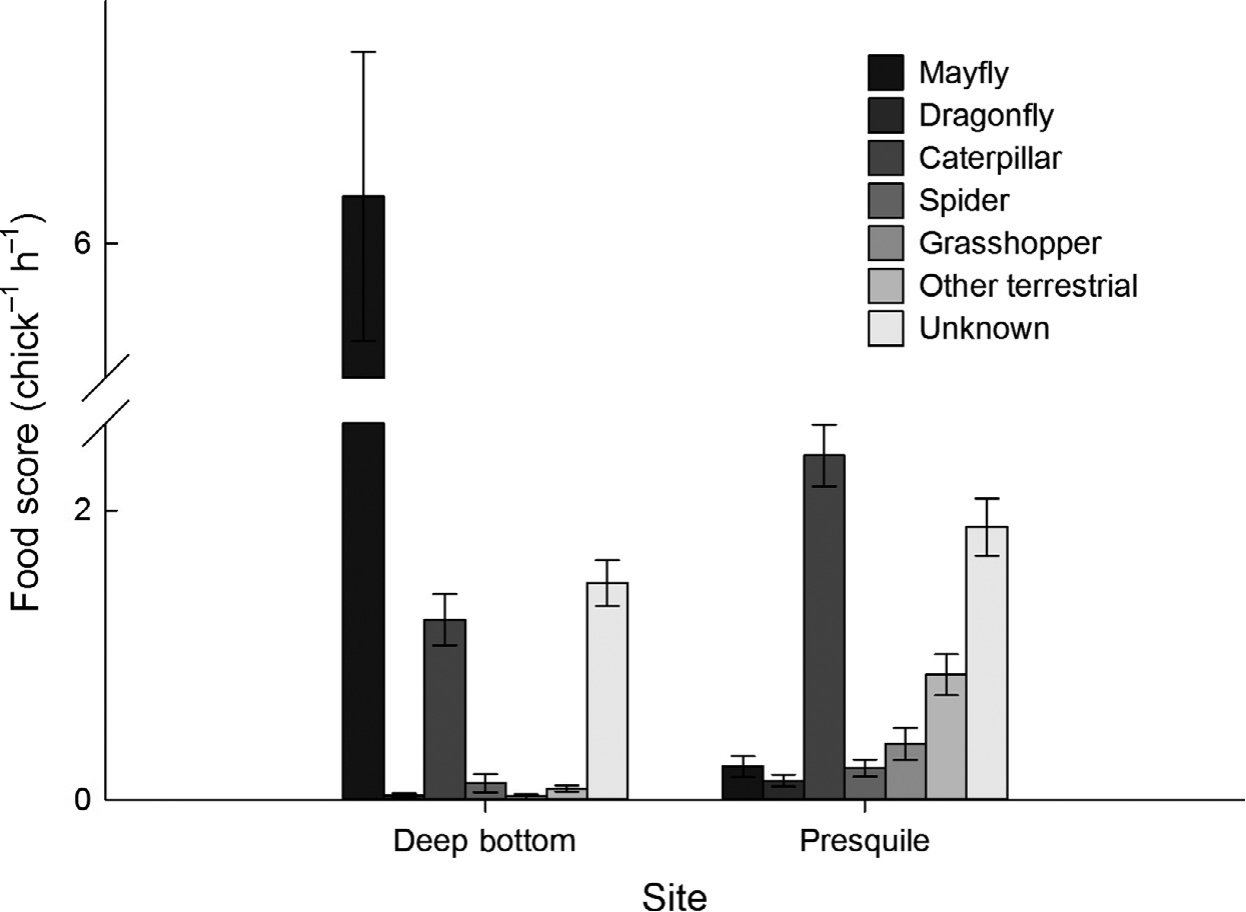
Mean food score of each prey type in nestling diet by site; mean ± 1 SE. Food score is an index of prey biomass where the size (relative to bird's bill) is multiplied by the quantity of each prey type.

Nestling diet differed greatly between sites. The number of parental visits did not differ between sites, although nestlings at Deep Bottom were provisioned more total prey (Table 1). Significantly, more aquatic prey were fed to nestlings at Deep Bottom, while more terrestrial prey were fed to nestlings at Presquile (Table 1). Nestlings at Deep Bottom were fed a greater amount of mayflies, while nestlings at Presquile were fed a greater amount of caterpillars (Fig. 3). Because mayflies are generally larger than caterpillars (mean dry mass = 24.1 ± 17.8 mg compared to 13.7 ± 21.3 mg for caterpillars), the larger overall food score at Deep Bottom could be due in part to the size differences in these prey items. Concordant with these results, mayflies and caterpillars comprised the greatest amount of food brought to nests at Deep Bottom and Presquile, respectively. Only rarely did parents bring spiders, grasshoppers, and other terrestrial prey to nests at Deep Bottom, whereas those noncaterpillar, terrestrial prey comprised 24% of the total food brought to nests at Presquile (Fig. 3).

**Table 1:** Provisioning and diet differences between our two study sites, Deep Bottom *n* = 59, Presquile *n* = 40. All values are the mean (chick^−1^·h^−1^) across all video recordings of prothonotary warbler nests. Mean ± 1 SD reported. All *P*‐values are from *t*‐tests except those with asterisks which did not meet normality assumptions. *P*‐values for these tests are from a Wilcoxon rank‐sum test

Variable	Deep bottom	Presquile	*P* value
Female visits	1.51 ± 0.812	1.47 ± 0.778	0.808
Male visits	1.29 ± 0.847	1.14 ± 0.744	0.370
Total food score	9.22 ± 4.14	5.92 ± 2.29	<0.0001
Aquatic food score	6.31 ± 4.35	0.40 ± 0.576	<0.0001*
Terrestrial food score	1.51 ± 1.63	3.71 ± 2.30	<0.0001*

### Nestling diet and prey availability

**Figure 4:**
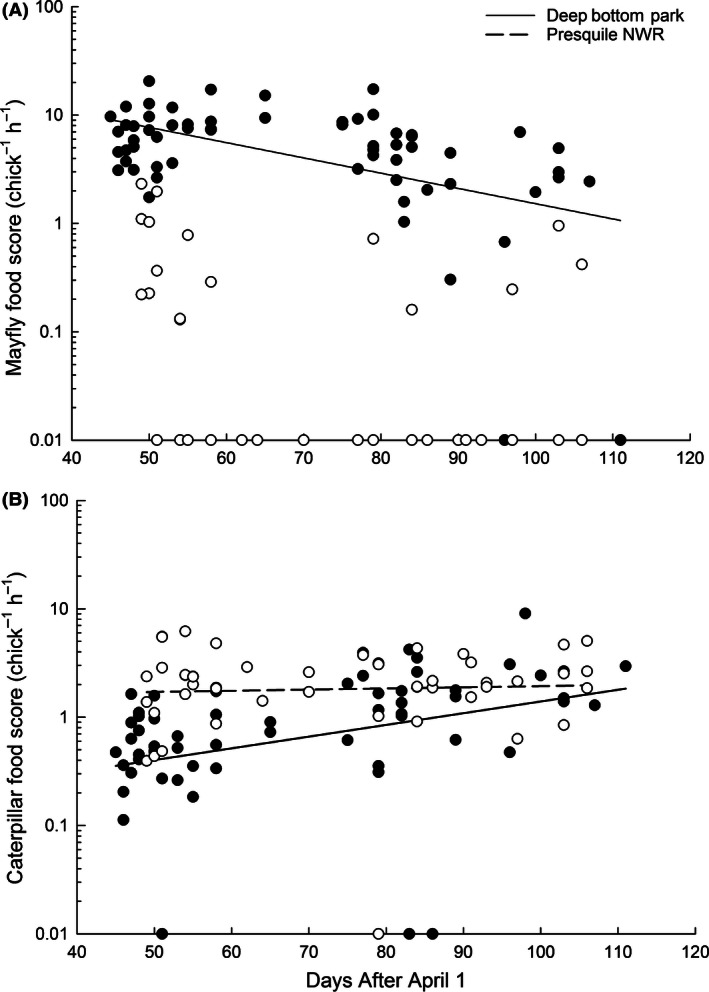
Total mayfly (A) and total caterpillar (B) food score brought to nestling prothonotary warblers by date at the two study sites; Deep Bottom (closed circle, solid line) and Presquile (open circle, dotted line). The Presquile mayfly line was not plotted because it did not meet the assumptions of normality due to the high number of zero values (see text). Nests that were fed no mayflies or caterpillars were given values of 0.01 because zero values do not appear on a log scale.

The MANOVA assessing prey type brought to individual nests demonstrated a significant multivariate effect whereby the amount of mayflies and caterpillars brought to the nests were different (Wilk's *λ* statistic = 0.183, *F*_10,206_ = 27.49, *P *<* *0.001). Specifically, prey items brought to nestlings varied with weekly mayfly biomass (*F*_2,103_ = 4.48, *P *<* *0.014), by site (*F*_2,103_ = 166.93, *P *<* *0.001) and by date (*F*_2,103_ = 14.04, *P *<* *0.001), and there was an interaction between site and date (*F*_2,103_ = 9.55, *P *<* *0.001). The types of prey brought to nestlings did not differ as a function of weekly caterpillar biomass (*F*_2,103_ = 2.47, *P *=* *0.090), although the relationship between weekly caterpillar biomass and caterpillar food score was positive. Multiple regression analysis indicated that 77% of the variation in the amount of mayflies brought to nestlings can be explained by weekly mayfly biomass (*β *= 0.120, *P *=* *0.035), site (Deep Bottom *β *= 0.342, *P *<* *0.0001), date (*β *= −0.004, *P *=* *0.0002), and the interaction between site and date (Deep Bottom *β *= −0.003, *P *=* *0.008). Significantly more mayflies were fed to nestlings at Deep Bottom than Presquile, and this amount declined throughout the season at Deep Bottom but stayed consistently low at Presquile (Fig. 4A). About 33% of the variation in caterpillar prey brought to nestlings was explained by site (Deep Bottom *β *= −0.088, *P *<* *0.0001), date (*β *= 0.003, *P = *0.002), and the interaction between site and date (Deep Bottom *β *= 0.002, *P *=* *0.047). Significantly more caterpillars were fed to prothonotary warbler nestlings at Presquile than Deep Bottom early in the season, but this difference decreased later in the season (Fig. 4B).

### Brood growth rate and body condition

**Figure 5:**
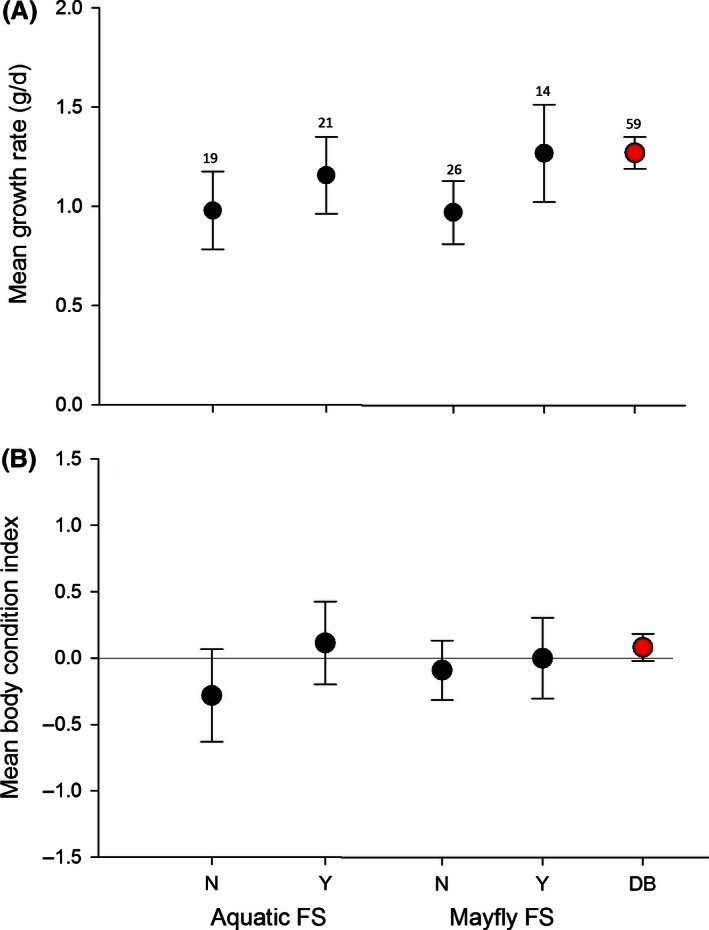
Mean growth rate (A) and body condition (B) for Deep Bottom (red) and Presquile (black). Presquile nest boxes were divided into categories representing whether or not they were fed mayfly prey or any aquatic prey. The figure represents means and 95% confidence intervals.

Mean nestling growth rate was higher at Deep Bottom (1.32 ± 0.39 g·day^−1^), where mayflies were very abundant, compared with Presquile (1.12 ± 0.34 g·day^−1^, *t* = −2.55, df = 85.07, *P *=* *0.013), where mayflies were less available. The base model for Presquile mean nestling growth rate included brood age (Table 2), where older nestlings had a slower growth rate than younger nestlings (Table 3). Adding a binomial variable for whether or not mayflies were fed to the nestlings resulted in the most supported model, where nests that were fed no mayflies had slower growth rates (Table 3). The model that included a binomial variable for whether or not aquatic prey were fed to nestlings performed similarly (Table 2). The parameter estimate confidence interval for brood age did not surround zero, suggesting this is a good predictor of nestling growth rate, but estimates for mayfly and aquatic food score include zero (Table 3). The most supported model for Deep Bottom nestling growth rate was the base model that included both brood age and male visits (Table 2) where mean growth rate significantly decreases with brood age and increases with male visits (Table 3). Adding mayfly or aquatic food scores did not improve model fit, although the models performed as well (∆ AIC_c_ < 2) as the base model. All parameter estimates surrounded zero, suggesting they are not strong predictors of nestling growth rate (Table 3). Mean nestling growth rate at Deep Bottom nests (1.30 ± 0.39 g·day^−1^) was not different from growth rate at Presquile NWR nests that were fed mayflies (1.27 ± 0.43 g·day^−1^, *P* = 0.99) or any aquatic prey (1.21 ± 0.44 g·day^−1^, *P* = 0.71) but was higher than nests that were not brought either (0.96 ± 0.44 g·day^−1^, *P* < 0.02) (Fig. 5A).

**Table 2:** Top models (∆AIC_c_ < 2) for factors predicting brood growth rate and body condition. Columns provide model notation, the number of estimable parameters (*K*), second‐order Akaike information criterion (AIC_c_), AIC_c_ differences compared to the top model (∆AIC_c_), and the adjusted R‐squared value for each model (Adj R^2^). FS = food score. FS_cat_ = categorical food score. Global models include factors known from previous studies to influence growth rate and condition (nestling age, brood size, and male and female visits per chick per hour) and do not include the aquatic or mayfly food score values. Base models include only the factors shown to influence growth or condition in this dataset based on the backwards stepwise regression analysis

	*K*	AIC_c_	∆AIC_c_	Adj *R* ^2^
**Presquile NWR Models**				
Growth rate				
Base model + mayfly FS_cat_	4	32.03	0.00	0.23
Brood age (Base model)	2	32.27	0.24	0.20
Base model + aquatic FS_cat_	4	33.63	1.60	0.19
Mayfly FS_cat_	2	39.09	7.06	0.05
Global model	6	39.13	7.10	0.19
Null (intercept only)	1	39.68	7.65	–
Aquatic FS_cat_	2	40.33	8.30	0.02
Body condition				
Aquatic FS_cat_	2	89.17	0	0.04
Ordinal date (Base model)	2	89.87	0.7	0.04
Null (intercept only)	1	90.01	0.84	–
Mayfly FS_cat_	2	91.51	2.34	0.02
Global model	5	93.74	4.57	0.05
**Deep Bottom Park Models**				
Growth rate				
Male visits + brood age (Base model)	3	21.26	0.00	0.18
Base model + aquatic FS	4	22.86	1.58	0.18
Base model + mayfly FS	4	22.88	1.60	0.18
Global model	6	25.88	4.62	0.18
Null (intercept only)	1	30.13	8.87	–
Aquatic FS	2	31.88	10.62	0.01
Mayfly FS	2	31.89	10.63	0.01
Body condition				
Ordinal date (Base model)	3	84.69	0	0.05
Global model	5	86.03	1.34	0.09
Null (intercept only)	1	86.24	1.55	–
Mayfly FS	2	87.18	2.49	0.01
Aquatic FS	2	87.29	2.6	0

**Table 3:** Parameter estimates from the top‐performing models predicting brood growth rate and body condition for each site separately. Columns provide parameter estimates, 95% confidence intervals, and *P* values from models. Mayfly FS and aquatic FS values are highly correlated with each other and are never included in models at the same time; estimates for these parameters are from models with these predictors and the italicized base model variables. Similarly, because mayfly and aquatic FS values were negatively correlated with ordinal date, they were not included in the same body condition models and parameter estimates are from models with these variables as the only predictors. PR = Presquile NWR and DB = Deep Bottom Park

	Parameter estimate	Confidence interval	*P*
Growth rate ‐ PR			
*Brood age*	−0.183	−0.30, −0.07	0.003
Mayfly FS_cat_ (0 mayflies)	−0.090	−0.20, 0.02	0.116
Aquatic FS_cat_ (0 aquatic prey)	−0.056	−0.16, 0.05	0.313
Body condition ‐ PR			
*Ordinal date*	−0.009	−0.02, 0.002	0.127
Aquatic FS_cat_ (0 aquatic prey)	−0.195	−0.42, 0.03	0.084
Mayfly FS_cat_ (0 mayflies)	−0.107	−0.37, 0.13	0.374
Growth rate ‐ DB			
*Male visits*	0.086	0.00, 0.17	0.056
*Brood age*	−0.119	−0.19, −0.04	0.003
Mayfly FS	−0.119	−0.37, 0.13	0.352
Aquatic FS	−0.122	−0.38, 0.13	0.342
Body condition – DB			
*Ordinal date*	−0.006	−0.01, 0.00	0.057
Mayfly FS	0.256	−0.20, 0.71	0.265
Aquatic FS	0.245	−0.21, 0.70	0.287

The most supported base model for mean body condition at both sites included ordinal date (Table 2) where later clutches were in poorer condition than earlier clutches (Table 3). Because mayfly and aquatic food score were positively correlated with ordinal date (mayflies declined throughout the season), we did not include these predictors in the same model. At Presquile, where mayflies were less available, a model including only aquatic food score ranked highest, but performed similarly as a model with only ordinal date and the null intercept‐only model (Table 2). At Deep Bottom, where mayflies were very abundant, models including mayfly or aquatic food score performed worse than those that included ordinal date or no predictors at all. There was no significant difference between sites for mean nestling body condition (Deep Bottom mean = 0.09 ± 0.55, Presquile = −0.11 ± 0.73, *t* = −1.55, df = 70.6, *P *=* *0.128), though Deep Boom nests, and those at Presquile that were fed aquatic prey tended to have more positive mean body conditions than those that were not fed aquatic prey (Fig. 5B).

### Site differences in reproductive parameters

Due to differences in growth rate between sites, we further examined site differences using a variety of fitness measures. The proportion of females that double brooded (Deep Bottom mean = 0.35, Presquile = 0.26, *x* ^2^ = 0.635, *P *=* *0.426) and average number of young fledged per female (Deep Bottom mean = 4.12 ± 3.0, Presquile = 3.97 ± 2.3, *t* = −0.26, df = 95, *P *=* *0.795) did not differ significantly between sites. The first clutch was initiated earlier at Deep Bottom (mean clutch initiation date = 3 May ± 2 days) compared with Presquile (13 May ± 3 days, *t* = 2.86, df = 58.47, *P *=* *0.006). In addition, nestlings at Presquile remained in the nest longer (11.9 ± 1.1 days) than nestlings at Deep Bottom (11.4 ± 1.3 days, *t* = 1.95, df = 106, *P *=* *0.053), which could be related to the slower growth rate observed at this site.

## Discussion

The effects of aquatic prey on reproductive success of riparian consumers have been largely ignored. Our study demonstrates that aquatic prey subsidies may influence nestling growth and condition in a passerine species. Variation in aquatic and terrestrial resources was observed in the prothonotary warbler nestling diet, and higher brood growth rates were found in a habitat with greater mayfly availability and use. Interestingly, at the site with low aquatic prey availability (Presquile), nests that were fed mayfly prey had higher brood growth rates than nests fed solely terrestrial prey, further demonstrating the importance of this resource.

The timing of peak caterpillar emergence did not overlap with the timing of greatest warbler nestling demand. Caterpillars were most available just prior to maximum egg production, and least available during the nestling period of both early and late clutches. While this pattern could be interpreted as poor timing when compared with other studies assessing caterpillar availability and avian nesting phenology (Van Noordwijk et al. 1995; Dias and Blondel 1996; Seki and Takano 1998; Naef‐Daenzer et al. 2000; Tremblay et al. 2003), it is not uncommon in other migrant species. Maziarz and Wesolowski (2010) observed that the date of maximum nestling demand for wood warblers, *Phylloscopus sibilatrix*, occurred 15–16 days after the peak in caterpillar abundance. Similarly, in a West Virginia forest, caterpillar density was higher earlier and later in the season, and lowest during the nestling period for red‐eyed vireos, *Vireo olivaceus* (Marshall and Cooper 2004). Our results are concordant with these studies, and seem to suggest that caterpillars could be an important energy resource for early season egg production in many migratory songbirds. A different trend was observed for aquatic prey where the maximum nestling demand occurred just after peak mayfly emergence, and mayflies remained abundant throughout the nestling period of both early and late clutches. Prothonotary warblers, similar to other migratory riparian species (Nakano and Murakami 2001), may time their breeding so that mayflies are available during the energetically demanding nestling period.

We observed a seasonal shift in diet to include more caterpillars as mayfly availability decreased (Fig. 4) at the site with higher mayfly availability. Likewise, other studies have shown that birds switch to alternative prey sources when preferred prey are less available (Blondel et al. 1991; García‐Navas and Sanz 2011); and similar seasonal diet shifts have been documented in the lesser spotted woodpecker, *Picoides minor* (Rossmanith et al. 2007) and wood warbler (Maziarz and Wesolowski 2010) from caterpillars to aphids and winged insects, respectively, as caterpillar abundance declined. However, no changes were observed in diet at the site with lower mayfly availability; nestlings were fed caterpillars consistently throughout the breeding season despite the seasonal variation in availability detected in our sampling. This site also had a more diverse diet, suggesting that prothonotary warblers opportunistically feed on a variety of prey types.

Despite the fact that warblers generally fed mayflies in relation to their abundance, our data indicate that mayfly resources may be sought out when in low abundance. Mayflies were not found in the vicinity of our nest boxes at Presquile, likely due to severe channel sedimentation and lack of suitable aquatic burrowing substrate, yet low numbers of mayflies were observed being fed to nestlings at this site (33% of boxes, 14 of 43), suggesting that parents travelled off territory to get these resources. Indeed, mayflies were observed in large numbers on the west side of the island (a distance ranging from 650 m to >1 km from the nest boxes) (Dodson and Moy pers. obs.) where there was suitable rocky substrate for *Hexagenia* spp. At the site with high mayfly availability, emergence near riverfront boxes occurred 2 weeks prior to that in the smaller creek, although we observed parents feeding mayflies at nest boxes prior to mayfly emergence in that location. This suggests that individuals may seek out valuable resources when they are nearby. Indeed, it has been documented that parent blue tits, *Cyanistes caeruleus*, will expand their foraging radius to acquire preferred caterpillar prey in habitats of low caterpillar availability (Tremblay et al. 2005). Although there is much to learn about extraterritorial movements in songbirds related to foraging, a recent study showed that we likely underestimate the area used by territorial songbirds during the breeding season (Streby et al. 2012). A study with Wilson's warblers, *Cardellina pusilla*, showed that males will leave their territory, often in pursuit of extra‐pair copulations, and frequently move 0.5 km and up to 2.5 km in search of these reproductive opportunities (Norris and Stutchbury 2001). Similar extraterritorial movements are possible for birds seeking foraging opportunities; however, they are expensive and not likely common during the demanding time of nestling feeding.

Other studies of riparian passerine species have observed the preference of aquatic prey in the nestling diet. Aquatic Diptera, particularly adult chironomids, were selectively fed to 8‐day‐old broods of yellow warbler nestlings, *Setophaga petechia* (Biermann and Sealy 1982), and Mengelkoch et al. (2004) observed 90–98% of the biomass fed to nestling tree swallows, *Tachycineta bicolor*, was of aquatic origin, primarily Odonates and aquatic Dipterans. Although our sampling efforts focused on mayflies in this study, the light traps also captured large numbers of other aquatic prey, primarily smaller aquatic species including Diptera (Nemotocera) and Trichoptera. It is possible that these smaller aquatic species were provisioned to prothonotary warbler nestlings, and we were unable to identify them in the videos due to their small size and coloration similar to the prothonotary warbler beak. As such, other types of aquatic prey, particularly chironomids, found as important components of the diet for other riparian species (Biermann and Sealy 1982; Mengelkoch et al. 2004), may be excluded from our results due to identification bias. Future studies of nestlings diet in this species may consider alternatives to video observation (i.e., crop flushing, collaring, or DNA in fecal material) to better understand the importance of smaller, less conspicuous, and readily available prey items.

Despite potential identification bias of some aquatic prey types, whether or not mayflies were fed to nestlings seemed to influence growth rate such that nestlings fed mayflies had faster mean growth rates than nestlings that were not. This relationship is not seen at our site with higher overall and aquatic prey availability, likely due to the superabundance of food (Tremblay et al. 2003). Reproductive performance only responds to increases in available food supply up to a certain threshold (Maziarz and Wesolowski 2010), beyond which parameters such as fledgling mass can be independent of food supply (Tremblay et al. 2005). In the habitat with greater food availability, mayfly abundance may have passed the saturation threshold (Maziarz and Wesolowski 2010), such that the relationship between nestling growth and the amount of mayfly prey in the diet was decoupled. However, at a site under the saturation threshold, the relationship between mayfly prey and reproductive parameters is more apparent; parents who were able to acquire mayflies had faster growing nestlings.

It is not surprising that nestling provisioning and diet would be related to measures of fitness, including growth rate and body condition (Tremblay et al. 2005). Ideally, parents feed nestlings resources that will promote rapid growth (to fledge as early as possible and avoid predation) and fledging at a larger mass (to promote postfledging survival). Our results indicate that this high‐quality resource may be of aquatic origin for the prothonotary warbler. To our knowledge, the only other bird study to assess the relationship between aquatic prey and fitness measures found more emerging aquatic prey led to higher pied flycatcher (*Ficedula hypolueca*) nestling survival rates along free‐flowing rivers compared to regulated rivers (Strasevicius et al. 2013). Aquatic prey has also been documented as an important resource for growth in riparian lizards; Sabo and Power (2002) found that growth rates were seven times higher in subsidized habitats during the early summer when emergence was highest. Additionally, within the watershed, lizard growth rates were positively correlated with the abundance of aquatic insects, further emphasizing the importance of aquatic subsidies for riparian predators. More studies are needed that assess fitness responses to spatial and temporal variations in emerging aquatic prey resources. This is particularly important because the timing of pulses in aquatic prey is regulated by temperature (Watanabe et al. 1999; Harper and Peckarsky 2006) and recent warming trends may lead to shifts in the timing of breeding in relation to prey abundance (i.e., a mismatch of prey supply and nestling demand) similar to those observed with caterpillars and the bird species that depend on them (Miller‐Rushing et al. 2013).

Although we found differences in nestling growth rate between habitats, diet differences do not appear to be affecting overall reproductive success, specifically the number of young fledged per female. Similarly, in the study of pied flycatchers that compared sites with high and low aquatic prey resources (Strasevicius et al. 2013), there was no difference in occupation rate, clutch size, or number of successfully hatched juveniles between habitats. In our system, we postulate that birds at the site with low mayfly availability (Presquile) make up for this deficit by spending more time in the nest; nestlings may benefit from a slower growth rate due to greater physiological development, greater flight ability, and better fledgling condition (Bosque and Bosque 1995). Nestlings could then leave the nest at an equivalent developmental stage and condition as nestlings from Deep Bottom where growth rates are faster. A tradeoff of this strategy is increased exposure to nest predators; however, in our study system, nest predation rates are low (boxes placed on poles over water) reducing the selective pressure for early fledging. We did find clutch initiation date to be earlier at the site with faster growth rates. Earlier breeding birds tend to be more successful as food availability generally decreases throughout the season (Daan et al. 1989; Bulluck et al. 2013), and studies have demonstrated that birds will delay initiation of nesting when food resources are low (Marshall et al. 2002; Strasevicius et al. 2013). As such, it appears that emerging aquatic insects may be key subsidies that are most important for early nesting birds.

As this was an observational study from only 1 year, we are cautious in our interpretation of the results and future work is recommended to understand the mechanisms responsible for nestling growth rates and to determine potential differences in nutritional content of caterpillars, mayflies, and other important aquatic prey items. Carbon‐to‐nitrogen ratios of invertebrates have been used to indicate relative amounts of chitin (Sullivan et al. 2014), a structural carbohydrate indigestible to birds. As such, it can be an indicator of food quality, where a greater proportion of chitin (or greater C:N) indicates lower quality (Sullivan et al. 2014). We found that mayflies had a lower (mean ± SD) C:N value (4.37 ± 0.28) than caterpillars (5.56 ± 0.07), which may in part explain the nutritional benefits of aquatic prey. In addition to C:N ratios, our results are also supported by potential nutritional differences in fatty acid content. Most recently, the literature has highlighted the dichotomy between omega‐3 long‐chain poly unsaturated fatty acid (LC‐PUFA) production between aquatic and terrestrial systems as a possible mechanism to explain food quality limitation in natural ecosystems. LC‐PUFAs are readily available in aquatic food webs as aquatic primary producers have high synthesis capacity (Hixson et al. 2015). However, terrestrial primary producers are not able to synthesize all of these fatty acids or their precursors, creating a fatty acid limitation in terrestrial‐based food (Hixson et al. 2015). Gladyshev et al. (2013) estimated that terrestrial carnivores may not be able to obtain sufficient amounts of omega‐3 highly unsaturated fatty acid (*ω*‐3 HUFA) consuming terrestrial‐based foods alone, and emergent aquatic insects may transport some *ω*‐3 HUFAs to terrestrial consumers, including riparian birds (Gladyshev et al. 2013). The clearest direct effect of *ω*‐3 HUFA limitation for an individual consumer is decreased growth (Twining et al. 2015), which could explain why nestlings at the site with few mayflies had slower growth rates. This suggests that relative differences in fatty acid content between terrestrial and aquatic resources could play an important role in nestling fitness in riparian species. Future work is recommended to determine differences in fatty acid content between caterpillars and mayflies.

Our findings support previous research that suggests aquatic resources are important subsidies for riparian species. Unlike previous studies that show changes in abundance, distribution, or migratory stopover refueling benefits related to pulses of emerging aquatic resources, our study is the first documentation of how aquatic subsidies in the nestling diet influence growth and condition in an avian species. Notably, aquatic resource availability and use did not seem to influence the annual reproductive success (number of young fledged) of individual birds. However, there could be “downstream” effects of differing food quality that were not measured in this study, such as fledgling survival and recruitment. Our results suggest that the interdependence between aquatic subsidies and riparian terrestrial consumers is crucial to understanding the breeding ecology of riparian species and that more study is needed in these complex systems.

## Conflict of Interest

None declared.
